# Identification and Characterization of Two Se6OMTs from *Stephania epigaea* Offer Novel Insights into the Biosynthetic Pathway of Cepharanthine

**DOI:** 10.3390/metabo15020092

**Published:** 2025-02-03

**Authors:** Jingyi Gan, Wenlong Shi, Qishuang Li, Xinyi Li, Xingyu Zhao, Junhao Tang, Ying Ma, Jian Wang, Shukun Gong, Xiaohui Ma, Juan Guo

**Affiliations:** 1 College of Traditional Chinese Medicine, Yunnan University of Chinese Medicine, Kunming 650500, China; ganjingyi01@163.com (J.G.); zxyrainyyy@126.com (X.Z.); tangjunhao@163.com (J.T.); 2 State Key Laboratory for Quality Ensurance and Sustainable Use of Dao-di Herbs, National Resource Center for Chinese Materia Medica, China Academy of Chinese Medical Sciences, Beijing 100700, China; shiwl031@163.com (W.S.); liqs556@163.com (Q.L.); lixinyi_cq@126.com (X.L.); xiaoma1110@126.com (Y.M.); jianwang2021@126.com (J.W.); 3 Key Laboratory of Yunnan Provincial Department of Education on Substance Benchmark Research of Ethnic Medicines, Yunnan University of Chinese Medicine, Kunming 650500, China; 4 State Key Laboratory of Southwestern Chinese Medicine Resources, Chengdu University of Traditional Chinese Medicine, Chengdu 611137, China; gongshukun@stu.cdutcm.edu.cn; 5 Dalian Institute of Marine Traditional Chinese Medicine, Dalian 116000, China

**Keywords:** cepharanthine, *Stephania epigaea*, 6OMT, biosynthesis, functional characterization

## Abstract

Background/Objectives: *Stephania epigaea* is a plant from the Menispermaceae family. Its root is an important traditional folk medicine, which is called Diburong in China. Diburong is rich in benzylisoquinoline alkaloids (BIAs), including cepharanthine, which has been demonstrated to exhibit significant anti-inflammatory, antiviral, antineoplastic, and anti-SARS-CoV-2 activities, as well as raising leukocytes. Cepharanthine is composed of (*R*)- and (*S*)-1-benzylisoquinoline alkaloid (1-BIA). (*S*)-norcoclaurine-6-O-methyltransferase (6OMT) is a rate-limiting enzyme in BIA biosynthesis. However, its role in the cepharanthine biosynthetic pathway, particularly with the (*R*) stereoisomer substrate, remains largely unexplored. This study aimed to identify Se6OMTs involved in the cepharanthine biosynthetic pathway and elucidate the *O*-methyltransferases (OMTs) responsible for the production of (*R*)- and (*S*)-stereoisomer BIAs. Methods: In this study, three OMTs were cloned from *S. epigaea* and functionally characterized using nine 1-BIAs of (*R*)- and (*S*)-configurations as substrates. Results: Two *O*-methyltransferases, Se6OMT1 and Se6OMT3, showed efficient catalytic activity at the C6 position of both (*R*)- and (*S*)-norcoclaurine. Furthermore, Se6OMT3 demonstrated high catalytic activity at the C7 and C4′ positions of other (*R*)- and (*S*)-configuration 1-BIAs, which resulted in the generation of multiple products. Conclusions: This study focused on 6OMT enzymes in *S. epigaea*, identifying Se6OMTs involved in the cepharanthine biosynthetic pathway, determining the OMTs involved in the production of (*R*)- and (*S*)-stereoisomer BIAs. This research provides valuable insights into the substrate promiscuity of Se6OMTs on (*R*)- and (*S*)-configured 1-BIAs in *S. epigaea* and highlights the genetic components necessary for the metabolic engineering and synthetic biology approaches to cepharanthine production.

## 1. Introduction

*Stephania epigaea* H.S.Lo, a member of the Menispermaceae family, is widely distributed in the southern regions of China [1]. Its root, known as Diburong in China, is a traditional folk medicine for the treatment of rheumatic arthralgia, fever, stomachache, diarrhea, bellyache, and fractures in Yunnan and Fujian provinces [2,3]. Diburong is rich in benzylisoquinoline alkaloids (BIAs), and over 60 BIAs are identified from *S. epigaea* [4]. These BIAs include bisbenzylisoquinoline (bisBIA), monobenzylisoquinoline (1-BIA), protoberberine, aporphine, morphine, and various other alkaloid types [4,5]. *S. epigaea* contains many compounds with pharmacological value, such as cepharanthine, a typical bisBIA among the main ingredients of *S. epigaea* [6]. Cepharanthine exhibits promising therapeutic effects, such as antituberculosis, antibacterial, and immuno-stimulating properties [7,8], and has been formulated into tablets for treating leukopenia in oncology patients [9]. Cepharanthine garnered significant attention in 2020 due to its potential therapeutic role in COVID-19 [10]. Subsequent research has confirmed its status as one of the most efficacious coronavirus inhibitors among clinically approved drugs [8,11], suggesting its potential utility against a broad spectrum of human pathogens [7,8]. *S. epigaea* is a significant source for the extraction of cepharanthine, yet this alkaloid is present at relatively low levels in the plant [12]. Elucidating the biosynthetic pathway of cepharanthine and leveraging synthetic biology techniques for its production represent significant strategies for expanding its availability and ensuring broader access to this valuable compound [13].

**Figure 1:**
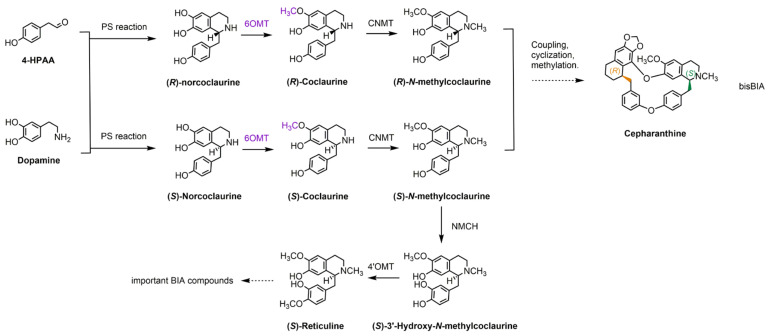
Proposed biosynthetic pathway of cepharanthine. Solid arrows indicate known pathways, whereas dashed arrows represent reactions that are either unknown or involve multiple steps. 6OMT, norcoclaurine 6-*O*-methyltransferase; CNMT, coclaurine *N*-methyltransferase; NMCH, *N*-methylcoclaurine 3′-hydroxylase; 4′OMT, 3′-hydroxy-*N*-methylcoclaurine 4′-*O*-methyltransferase; PS reaction, Pictet–Spengler reaction.

The upstream pathway of cepharanthine is similar to that of other BIAs. BIAs originate from a common biosynthetic pathway that begins with the Pictet–Spengler condensation of *L*-tyrosine derivatives, leading to the formation of (*S*)-norcoclaurine, which is the first intermediate compound in this pathway. Norcoclaurine, catalyzed by enzymes such as 6OMT, sequentially gives rise to compounds such as coclaurine and *N*-methylcoclaurine (NMC) (Figure 1). However, there have been few reports on the enzymes responsible for the formation of (*R*)-1-BIA in the upstream pathway.

Despite the recent availability of genomes from several Menispermaceae plants capable of producing cepharanthine, the understanding of their biosynthetic pathways remains incomplete [11]. Although the biosynthetic pathway of cepharanthine is not fully elucidated, it is hypothesized to originate from *N*-methylcoclaurine (NMC) [14,15,16,17]. The pathway likely involves the coupling of two 1-BIA molecules with a C-O phenol, followed by cyclization, the formation of a methylenedioxy bridge, and methylation, ultimately resulting in cepharanthine, which consists of one molecule of (*R*)-1-BIA and one molecule of (*S*)-1-BIA (Figure 1); the upstream pathway of cepharanthine is similar to other BIAs [18]. BIAs are derived from a common biosynthetic pathway that begins with the Pictet–Spengler condensation of *L*-tyrosine derivatives, leading to the formation of (*S*)-norcoclaurine, the first intermediate compound in this pathway [19]. 6OMTs catalyze the conversion of norcoclaurine into coclaurine [20] (Figure 1). However, there have been few reports on the enzymes responsible for the formation of (*R*)-1-BIA in the upstream pathway.

6OMT, a cation-independent enzyme classified as a type I OMT [21], initiates BIA biosynthesis by methylating (*S*)-norcoclaurine at the C6 position using *S*-adenosyl-*L*-methionine (SAM), resulting in the formation of (*S*)-coclaurine. As a rate-limiting enzyme, 6OMT plays a pivotal role in BIA biosynthesis [22]. First identified in *Coptis japonica* [23], 6OMT has been the subject of several recent studies within the Menispermaceae family; however, the catalytic function of 6OMT with different configurations of substrates remains incompletely understood [20,24]. Considering that the biosynthetic pathway of cepharanthine involves the coupling of *R*-type and *S*-type monomers, elucidating the catalytic function of 6OMT in different configurations of 1-BIA within its upstream pathway is essential. To clarify the methylation modification of (*R*)-1-BIA within the cepharanthine biosynthetic pathway, we screened all *OMTs* from the transcriptome of *S. epigaea* to construct a library of *OMT* genes. We then selected three *6OMT* candidates and assessed their catalytic capabilities towards both (*R*)- and (*S*)-norcoclaurine through enzymatic reaction assays, thereby elucidating the C6 methylation process in the (*R*)-stereoisomer of the cepharanthine biosynthetic pathway. Furthermore, we expanded the substrate scope and conducted additional research on the substrate promiscuity of these three 6OMTs. Collectively, this study lays the groundwork for the elucidation and heterologous reconstruction of the cepharanthine biosynthetic pathway and contributes to the expansion of the substrate range for 6OMTs.

## 2. Materials and Methods

### 2.1. Plant Materials, Chemicals, and Strains

The *S. epigaea* used in this experiment was collected from the Yunnan University of Chinese Medicine in Kunming, Yunnan Province, China, in 2022. Post-collection, the samples were washed; separated into roots, stems, and leaves; flash-frozen with liquid nitrogen; and stored at −80 °C. The (*S*)-*N*-methylcoclaurine and (*R*)-*N*-methylcoclaurine standards were synthesized by WuXi LabNetwork (Shanghai, China). The norcoclaurine and coclaurine were sourced from Baoji Herbest Bio-Tech Co., Ltd., (Baoji, China), and after chiral separation, (*S*)-norcoclaurine, (*R*)-norcoclaurine, (*S*)-coclaurine, and (*R*)-coclaurine were obtained. Additional standard products were provided by Shanghai Yuanye Bio-Technology Co., Ltd., (Shanghai, China). These standards were dissolved in methanol or DMSO to prepare 10 mM solutions used as substrates for in vitro enzymatic reactions. The *S*-adenosine-*L*-methionine was purchased from Solarbio (Beijing, China), with all commercial chemicals being of ≥95% purity. The acetonitrile used in the compound detection experiments was of a chromatographically pure grade and sourced from Merck (Darmstadt, Germany).

The pET-28a plasmid was supplied by our laboratory. The strains *Escherichia coli* Trans1-T1 (*E. coli* Trans1-T1) and BL21(DE3) [*E. coli* BL21(DE3)], used for cloning and in vitro expression, were obtained from TransGen Biotech (Beijing, China).

### 2.2. RNA Extraction and Transcriptome Sequencing

Trizol was utilized for RNA extraction from *S. epigaea* tissue. The concentration, purity, and integrity of the RNA were assessed using agarose gel electrophoresis and a micro spectrophotometer. Following sequencing, gene annotation was conducted using databases including GO [25], KEGG [26], PATHWAY [26], UniProt [27], COG [28], Nr [29], and Pfam [30] for an in-depth analysis to identify highly expressed methyltransferases.

### 2.3. Analysis and Cloning of St6OMTs Genes

Methyltransferases (MTs) play a crucial role in the methyl formation of BIAs, a structurally diverse group of plant-specialized metabolites. To identify and characterize the 6OMT enzymes involved in the biosynthetic pathway of cepharanthine in *S. epigaea*, we utilized the transcriptome data from this plant. Based on the transcriptome annotation, we established a database comprising 128 OMTs for subsequent genetic screening.

We selected the OMTs and previously reported 6OMTs to construct a phylogenetic tree using MEGA11, amino acids were aligned with ClustalW, and a phylogenetic analysis was performed using the neighbor-joining method with 1000 bootstrap replicates.

The cDNA synthesis was performed using the PrimeScript™ IV 1st Strand cDNA Synthesis Mix Kit (Takara, Tokyo, Japan). The open reading frame sequences of the SeMTs were amplified with PrimeSTAR^®^ Max DNA Polymerase (Takara) with the primers listed in Supplementary Table S2, and cloned into the pET-28a vector. The recombinant plasmid was transformed into *E. coli* Trans1-T1, and the successful ligation of the target fragment was confirmed using 2×EasyTaq^®^ PCR SuperMix (TransGen Biotech, Beijing, China) and sequencing verification. The correctly sequenced plasmid was extracted using the Plasmid Extraction Kit (Magen Biotech, Guangzhou, China) and stored at −20 °C.

### 2.4. Expression in E. coli BL21(DE3) and Recombinant Protein Purification

The extracted plasmid was transferred into *E. coli* BL21(DE3)-competent cells (TransGen Biotech) and plated on LB solid medium containing 100 μg/mL of ampicillin, then cultured overnight at 37 °C. Single colonies were then inoculated into 100 mL of LB liquid medium supplemented with 100 μg/mL of ampicillin and grown at 37 °C with shaking at 200 rpm until the OD600 reached 0.6–1.0. Isopropyl *β*-D-thiogalactoside (IPTG) was added to a final concentration of 0.3 mmol/L, and the culture was incubated at 16 °C with shaking at 160 rpm for 12–16 h. The cells were harvested by centrifugation at 8000× *g* for 5 min and resuspended in 5 mL of Tris-HCl buffer (100 mmol/L, pH 7.5). Phenylmethylsulfonyl fluoride (PMSF) was added to a final concentration of 0.1 mol/L, and the crude protein was obtained by sonication on ice using a Branson Digital Ultrasonic Amplifier (Danbury, CT, USA) at 60% amplitude for 8 min (5 s on, 5 s off). The lysate was centrifuged at 4 °C and 10,000× *g* for 30 min, and the supernatant was applied to pre-equilibrated Ni-NTA resins (TransGen). The resin was washed with 4 mL of imidazole buffer at varying concentrations, and a 200 mM imidazole elution fraction was collected. The protein concentration and purity were assessed using an Ultra Trace UV Spectrophotometer (Implen GmbH, Munich, Germany) and 10% SDS–polyacrylamide gel electrophoresis. The purified protein was mixed with 50% glycerol and stored at −80 °C.

### 2.5. Functional Characterization of the Recombinant St6OMTs

The purified protein was tested for 6OMT activity in a 300 μL solution system containing 100 mmol/L of Tris-HCl (pH 7.5), 0.33 mM of substrate, 0.17 mM of SAM, and 50 μg of purified protein. The proteins extracted from the *E. coli* BL21(DE3) strain containing the pET-28a vector alone served as negative controls. The reaction mixture was incubated at 30 °C with shaking at 200 rpm for 3 h. Subsequently, 10 μL of ammonium hydroxide and 500 μL of ethyl acetate were added to the system for product extraction. After centrifugation, the supernatant was collected, dried, and redissolved in methanol for further testing.

### 2.6. UPLC-QTOF-MS Analysis

The enzymatic reaction products of 6OMT were detected using UPLC-QTOF-MS (Waters Technologies, Milford, MA, USA). The analysis was performed on a T3 column (Waters Technology, 2.1 × 100 mm, 2.7 μm particle size) at 38 °C with an injection volume of 1 μL. The mobile phase consisted of 0.1% formic acid in water (solvent A) and acetonitrile (solvent B) at a flow rate of 0.4 mL/min. The linear gradient elution was programmed as follows: 95% to 70% solvent A from 0 to 6 min, 70% to 10% solvent A from 6 to 9 min, 10% to 95% solvent A from 9 to 9.5 min, and 95% solvent A from 9.5 to 11 min.

The UPLC system was coupled to a Waters Xevo G2-S QTOF mass spectrometer (Waters Technologies, Milford, MA, USA), equipped with an electrospray ionization (ESI) source. The instrument was operated in positive ion mode, with full-scan monitoring over the *m*/*z* range of 50–800. The following parameters were optimized: capillary voltage of 0.5 kV, sample cone voltage of 40 V, extraction cone voltage of 4 V, source temperature of 100 °C, desolvation temperature of 300 °C, and desolvation gas flow rate of 800 L/h. The trap collision energy for the low-energy function was set to 6 eV, while for the high-energy function, it ranged from 30 to 50 eV [31]. The data acquisition and analysis were performed using MassLynx V4.1 software.

## 3. Results

### 3.1. Screening, Cloning, and Analysis of 6OMTs from the Transcriptome of S. epigaea

**Figure 2:**
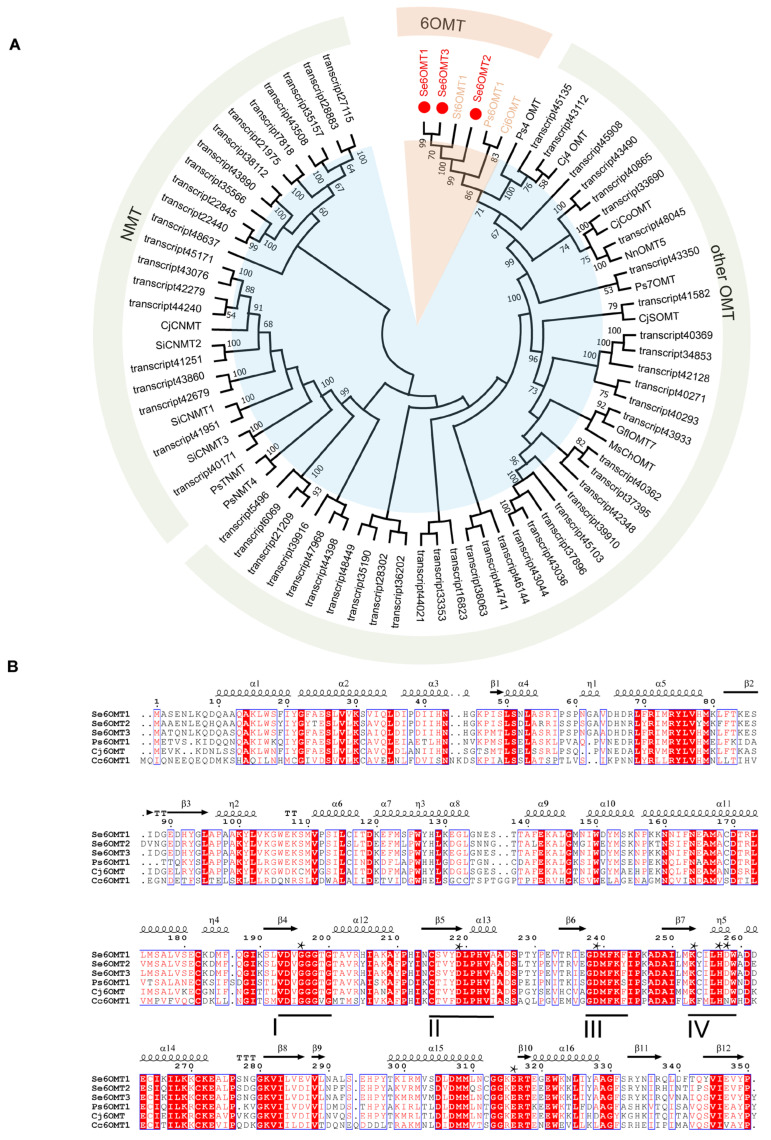
Phylogenetic analysis and amino acid sequence alignment of candidate SeOMTs with other functional characterized OMTs. (**A**) Seventeen OMT protein sequences retrieved from GenBank were used to construct the phylogenetic tree. The figure presents a phylogenetic tree and amino acid sequence alignment of candidate SeOMTs. It includes the 64 SeOMTs discussed in this study, with the three Se6OMTs identified here highlighted in red circles. Abbreviations and GenBank accession numbers are provided in Supplementary Table S4. (**B**) The sequence alignment of *P. somniferum* 6OMT (Ps6OMT1), *C. japonica* (Cj6OMT), and *C. chinensis* (Cc6OMT1) with *S. epigaea* 6OMTs. Conserved motifs I–IV are underlined, and asterisks indicate conserved residues, including BIA binding sites (H256, D257), SAM binding sites (G195, D218, D238, K252), and a critical residue (E315).

We identified 128 OMT genes annotated in the transcriptome of *S. epigaea*, utilizing these genes as a library for screening potentially functional genes. After excluding genes with FPKM values below 10 and removing those with high sequence similarity, we conducted a comparative analysis of Se6OMTs alongside functionally characterized MTs from other species, including 6OMT, 4′OMT, 7OMT, RNMT, CNMT, and TNMT (Figure 2A). Based on the results of a phylogenetic tree analysis, we selected three OMTs that clustered with other 6OMTs for further identification. The nucleic acid sequences of Se6OMT1-3 are presented in Supplementary Table S1.

Se6OMT1, Se6OMT2, and Se6OMT3 encode 350, 351, and 351 amino acids, respectively. These three proteins have molecular masses of approximately 43 kDa and exhibit amino acid sequence identity values ranging from 81% to 93%, with the highest identity observed between Se6OMT1 and Se6OMT3. Multiple sequence alignments indicated that key residues in Se6OMTs are highly conserved (Figure 2B). Notably, these key features include the S-adenosylhomocysteine–S-adenosylmethionine (SAH/SAM) interaction region and four conserved motifs (labeled I–IV), which are characteristic of the SAM binding site in plant SAM-dependent methyltransferases [32].

The multiple sequence alignment of *Papaver somniferum* 6OMT1 (Ps6OMT1) [33], *C. japonica* 6OMT (Cj6OMT) [23], *Coptis chinensis* 6OMT1 (Cc6OMT1) [34], and Se6OMTs revealed that all of these enzymes possess the conserved motifs I–IV [32] (Figure 2). The residues within these motifs that directly interact with SAM, including G195 in motif I, D218 in motif II, D238 in motif III, and K252 in motif IV, were found to be conserved among the seven SeOMTs examined [19]. The residues involved in BIA substrate binding, H256 and D257, along with the key catalytic residue, E315, were conserved among the three SeOMTs examined [19]. Furthermore, a highly conserved glycine-rich ‘GxGxG’ sequence within the SAM-binding motif I was identified in SeOMT1, SeOMT2, and SeOMT3. These findings provide a foundation for the subsequent functional characterization of the three OMTs.

### 3.2. Functional Characterization of Se6OMTs Towards (R)- and (S)-Norcoclaurine

Three recombinant Se6OMTs were cloned into the pET-28a vector and expressed in *E. coli BL21*(*DE3*). After induction with IPTG, the proteins were purified using a Ni column. The SDS-PAGE analysis confirmed the soluble expression of Se6OMTs with molecular weights close to the predicted 43 kDa (Figure S1). The physicochemical properties of the Se6OMTs are detailed in Table S3.

Cepharanthine is synthesized via a C-O coupling reaction involving one molecule of (*S*)-1-BIA and one molecule of (*R*)-1-BIA [14,15,35]. However, the actions of 6OMT within the upstream 1-BIA pathway in *S. epigaea* remain largely unexplored, especially concerning the methylation process of (*R*)-1-BIA. Therefore, in vitro enzymatic reactions were conducted using recombinant proteins, with SAM as the methyl donor and (*S*)- and (*R*)-norcoclaurine as substrates to assess their catalytic functions. Proteins extracted from *E. coli* BL21(DE3) containing the pET-28a vector served as negative controls. The results indicated that Se6OMT1 and Se6OMT3 exhibited distinct activities towards norcoclaurine, while Se6OMT2 showed no activity on the tested intermediates.

**Figure 3:**
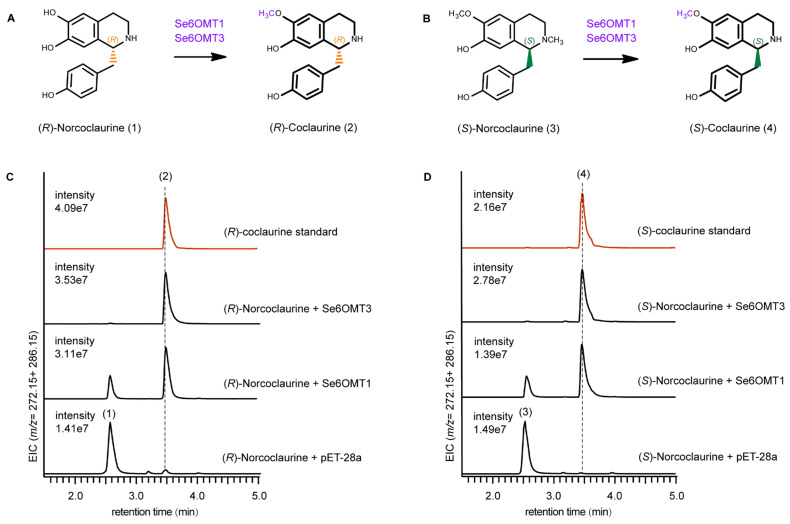
In vitro enzyme assay of Se6OMTs using (*R*)- and (*S*)-norcoclaurine as substrates. (**A**,**B**) Enzymatic reaction catalyzed by Se6OMT1 and Se6OMT3. The functional Se6OMTs are indicated above the arrows in the catalytic diagram, highlighted in purple. (**C**,**D**) Extracted ion chromatograms of the catalytic products from Se6OMT1 and Se6OMT3. The numerical labels on the chromatographic peaks correspond to the identifiers listed on the substrates and products in the reaction diagrams. The red line represents the standards (*R*)- and (*S*)-coclaurine.

For product identification and verification, UPLC-QTOF-MS was utilized to determine the molecular weights of the products, compare their retention times, and assess their structural characteristics. Se6OMT1 and Se6OMT3 catalyzed the methylation at the C6 position of (*R*)-norcoclaurine (1) to produce (*R*)-coclaurine (2), as evidenced by retention times and MS spectra when compared to authentic standards. Product 2, with a mass-to-charge ratio (*m*/*z*) of 286.16, showed a mass increase of 14 units compared to substrate 1 (*m*/*z* 272.14) and was identified by comparison with an authentic standard. The retention time and fragmentation pattern in the secondary mass spectrometry of this compound were consistent with the (*R*)-coclaurine standard (Figure S2). In other words, Se6OMT1 and Se6OMT3 could catalyze the conversion of (*R*)-norcoclaurine to (*R*)-coclaurine, with Se6OMT3 exhibiting higher activity than Se6OMT1 in O-methylation at the C6 position on (*R*)-norcoclaurine, as indicated by product abundance (Figure 3C).

Se6OMT1 and Se6OMT3 also displayed 6-*O*-methylation activity with (*S*)-norcoclaurine as the substrate. The mass spectrometry (Figure S2) and retention times matched the standard for (*S*)-coclaurine, with no significant difference in peak intensity. However, Se6OMT1 showed the residual substrate in the reaction, while Se6OMT3 nearly eliminated it, indicating higher catalytic efficiency for Se6OMT3 in the *O*-methylation of the C6 position on (*S*)-norcoclaurine (Figure 3D). Se6OMT1 and Se6OMT3 efficiently catalyzed the conversion of both (*R*)- and (*S*)-norcoclaurine to their respective (*R*)- and (*S*)-coclaurine forms, showing no substrate preference.

### 3.3. Functional Characterization of Se6OMT3 on Other Seven 1-BIAs Substrates

Seven representative BIA compounds, in addition to norcoclaurine, including the (*R*)- and (*S*)-configurations of coclaurine, NMC, (*S*)-3′-hydroxy-*N*-methylcoclaurine [(*S*)-HNMC)], (*S*)-reticuline, and (*R*)-norarmepavine (Figure S3), which contain hydroxyl groups at the C7, C3′, and C4′ positions and provide abundant sites for *O*-methylation, were used as substrates to characterize the catalytic promiscuity of Se6OMTs, with SAM serving as the methyl donor. The products of the enzyme assays on these substrates were qualitatively analyzed through in vitro experiments and UPLC-QTOF-MS.

**Figure 4:**
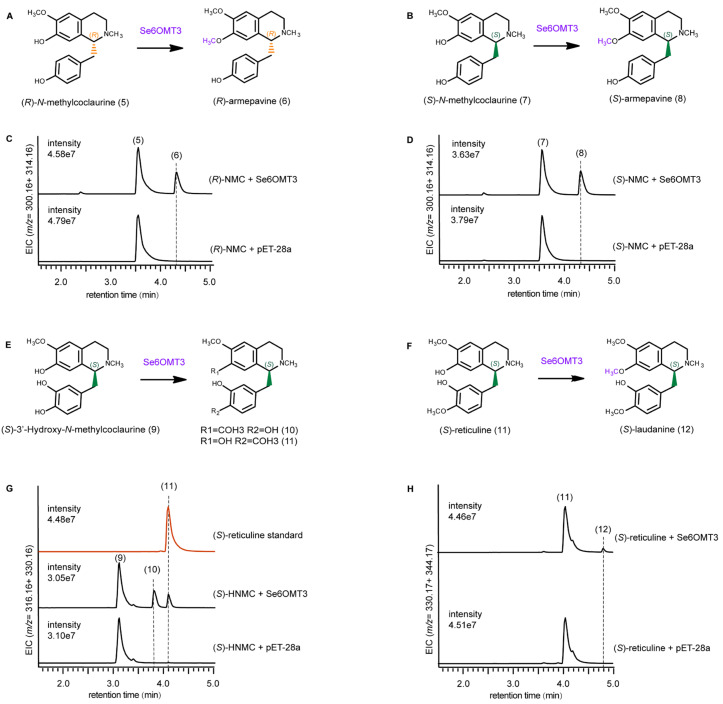
Substrate promiscuity of St6OMT3 using multiple 1-BIAs as substrates. (**A**,**B**,**E**,**F**) *O*-methylation reactions catalyzed by St6OMT3. The substrates and products of St6OMT3 reactions are depicted. (**C**,**D**,**G**,**H**) Extracted ion chromatograms correspond to reactions with (*R*)- and (*S*)-*N*-methylcoclaurine, (*S*)-3′-hydroxy-*N*-methylcoclaurine, and (*S*)-reticuline as the substrate, respectively. The numeric labels attached to the chromatographic peaks match the identifiers shown on the substrates and products in the reaction diagrams. Substrate and product identifiers: (*R*)-NMC, (*R*)-*N*-methylcoclaurine (5); (*S*)-NMC, (*S*)-*N*-methylcoclaurine (7); (*S*)-HNMC, (*S*)-3′-Hydroxy-*N*-methylcoclaurine (9); (*S*)-7-methyl-3-hydroxy-*N*-methylcoclaurine (10); (*S*)-reticuline (11).

Based on a comparison of their retention times and mass spectra with those of the reference standards, Se6OMT3 catalyzed the methylation at the C7 position of (*R*)-NMC and (*S*)-NMC, producing (*R*)-armepavine and (*S*)-armepavine, respectively, without showing a preference for either (*R*)- or (*S*)-NMC (Figure 4C,D). The mass spectrometry data analysis of both the reaction products and substrates revealed an additional peak at *m*/*z* 206 in the product fragments, indicating a 14-unit increase in the *m*/*z* value of the isoquinoline component, suggesting methylation at the hydroxyl group on the C7 position (Figure S2). Similarly, Se6OMT3 also methylated (*S*)-HNMC at the C7 position, with peak 10 proposed to be (S)-7-methyl-3-hydroxy-N-methylcoclaurine (10) (Figure 4G) based on previous studies [36].

Using (*S*)-reticuline (11) as a substrate, Se6OMT3 selectively methylated at the C7 position, yielding (*S*)-laudanine (12) (Figure 4H). Additionally, Se6OMT3 catalyzes methylation at the C4′ position of (*S*)-HNMC to produce (*S*)-reticuline. The abundance of this product was slightly higher than that of the Se6OMT3-mediated C7 methylation of (*S*)-reticuline but lower than the C7 methylation of NMC and (*S*)-HNMC by Se6OMT3 (Figure 4G). The mass spectra of fragment ions are presented in Figure S2. Se6OMT3 exhibits a broader substrate range for 1-BIAs, accepting several BIA pathway intermediates as substrates, including the C7 positions of (*R*)-NMC, (*S*)-NMC, (*S*)-reticuline, and (*S*)-HNMC, and the C4′ positions of (*S*)-HNMC, demonstrating high substrate promiscuity and catalytic site promiscuity. In contrast, no products were detected in reactions containing Se6OMT1 and Se6OMT2 with these substrates. Overall, these OMTs provide essential genetic elements essential for the biosynthesis of a variety of BIAs.

## 4. Discussion

BIAs are essential secondary metabolites in plants, playing a crucial role in drug development [37]. Cepharanthine, a notable bisBIA, is an important member of the BIA family [12]. The recent coronavirus outbreak has brought cepharanthine into focus due to its reported strong antiviral effects against SARS-CoV-2 [8,10,38]. Understanding the biosynthetic pathway of cepharanthine offers new strategies for its production. Cepharanthine is biosynthesized through a C-O coupling reaction between one molecule of (*S*)-1-BIA and one molecule of (*R*)-1-BIA [14,15,35]. However, the action of 6OMT in the upstream 1-BIA pathway in *S. epigaea* remains largely unexplored, particularly regarding the methylation of (*R*)-1-BIA. This study analyzed the transcriptome of *S. epigaea*, which contains cepharanthine, identifying all OMTs and pinpointing three 6OMTs through a phylogenetic analysis, whereby enzymes with similar functions generally cluster together, providing a reference for functional characterization. Notably, Se6OMT1 and Se6OMT3 efficiently catalyzed the conversion of (*R*)-norcoclaurine and (*S*)-norcoclaurine to their respective coclaurine configurations. This represents the first report of 6OMTs in *S. epigaea*, with particular significance in characterizing the function of Se6OMT on (*R*)-type 1-BIA. This research establishes a foundational study for the biosynthesis of cepharanthine.

Methyltransferases (MTs), which are particularly abundant in the BIA biosynthetic pathway, play a crucial role in increasing molecular diversity through post-modification [36]. 6OMT is recognized as the initial MT and a rate-limiting enzyme within this pathway [19]. While most of the research on MTs has focused on (*S*)-enantiomeric or racemic substrates [24], the distinctive (*R*)-configuration BIA and its biosynthetic routes have been minimally explored. The biosynthesis of cepharanthine necessitates the involvement of (*R)*-1-BIA. Our research findings indicate that Se6OMT1 and Se6OMT3 exhibit comparable catalytic activity and efficiency towards both (*S*)- and (*R*)-norcoclaurine. This suggests that plants have the capacity to form both *S*- and *R*-configured coclaurines indiscriminately, at least during the C6 methylation step. In terms of catalytic efficiency, Se6OMT3 consistently outperforms Se6OMT1, nearly converting all substrate to product, demonstrating different catalytic efficiencies between these two Se6OMTs.

The methylation step in the BIA biosynthetic pathway is catalyzed by methyltransferases that show significant substrate promiscuity [39]. The promiscuity of 6OMT is widespread; CyOMT7 (6OMT) efficiently catalyzes the methylation at the C6 position of (*S*)-norlaudanosoline and also accepts (*S*)-HNMC, (*S*)-tetrahydrojatrorrihizine (THJ), and quaternary protoberberine alkaloids as substrates for methylation at the C7, C2, and C3 positions, respectively [36]. Similarly, GFLOMT1 efficiently catalyzes the C6 position of norlaudanosoline and also acts on the C7, C3′, and C4′ positions of the BIA skeleton [40]. This promiscuity enables the synthesis of a diverse array of compounds but also leads to significant by-product formation during the heterologous production of specific compounds. In our study, we investigated the catalytic promiscuity of Se6OMT1 and Se6OMT3 using various 1-BIA substrates. Se6OMT1 showed a high degree of catalytic specificity, exclusively catalyzing the *O*-methylation at the C6 position of (*S*)- and (*R*)-configured norcoclaurine. In contrast, Se6OMT3 displayed broader promiscuity, utilizing *S*- and *R*-configured NMC as substrates for O-methylation at the C7 position, in addition to norcoclaurine. Furthermore, Se6OMT3 also catalyzed weaker *O*-methylation reactions at the C7 or C4′ positions when (*S*)-reticuline and (*S*)-HNMC were used as substrates. The amino acid sequences of enzymes Se6OMT1 and Se6OMT3 share 93% similarity. However, differences in their C-terminal and N-terminal regions suggest potential functional divergence. Further mutagenesis studies are required to elucidate the structural basis of these differences. *N*-methylation of the substrate affects the overall hydrophobicity, spatial volume, and conformation of the molecule, thereby influencing enzyme functions [41]. The distinct catalytic properties of Se6OMT1 and Se6OMT3 suggest their suitability for different applications. In synthetic biology, Se6OMT1 can be used as a functional component when specific 6OMT activity is desired, while Se6OMT3 can act as a catalytic component to produce a structurally diverse range of products, indicating a potential for the development of this enzyme into a broadly useful tool for methyltransferase reactions. Although previous studies have explored the catalytic mechanisms of methyltransferases in the BIA biosynthetic pathway, it has been demonstrated that M111 in *Thalictrum flavum* scoulerine 9-*O*-methyltransferase (S9OMT) is a key residue for the 9-*O*-methylation of scoulerine, and that mutating M111 to alanine leads to an expanded substrate scope towards various 1-BIA substrates [42]. In this study, SeOMT3, which effectively catalyzes the methylation at the C6, C7, and C4′ positions of 1-BIA substrates, was found to possess the M111 residue; the mechanisms governing substrate promiscuity and specificity for 6OMTs still require further elucidation through mutation or other structural biology research.

## 5. Conclusions

In summary, this study concentrated on *S. epigaea*, a plant rich in cepharanthine, to identify and characterize the 6OMT enzymes involved in the biosynthetic pathway of cepharanthine. The study elucidated the catalytic functions of these enzymes on (*R*)- and (*S*)-configured norcoclaurine, and through multi-substrate experiments revealed that Se6OMT1 exhibits substrate specificity, whereas Se6OMT3 demonstrates broader substrate promiscuity. Although the biosynthetic pathway of cepharanthine remains largely unexplored, the identification of Se6OMTs in this study provides a foundation for the further elucidation of the unknown pathways and provides a reference for the screening of candidate genes. This work is anticipated to offer significant insights into the genetic components necessary for the heterologous production of cepharanthine, as well as for the synthesis of other (*R*)- and (*S*)-configured BIAs.

## Data Availability

The data presented in this study are available in article and Supplementary Material.

## Supplementary Materials

The following supporting information can be downloaded at https://www.mdpi.com/article/10.3390/metabo15020092/s1: Figure S1. SDS-PAGE analyses were conducted on the purified proteins of St6OMT1-3 from the induced expression of the recombinant plasmid. Figure S2. High-resolution MS/MS spectrum fragmentation of products catalyzed by Se6OMTs. Figure S3. Seven compound structures of 1-BIAs were tested as potential substrates of Se6OMTs. Table S1. The full-length nucleic acid sequences of *Stephania epigaea* OMTs in this study. Table S2. The primer sequences for cloning Se6OMTs-pET-28a. Table S3. The physical and chemical properties of Se6OMTs in this study. Table S4. Abbreviations and GenBank accession numbers for functionally characterized plant OMTs used for the phylogenetic analysis.
