# Supplementary material for: Identification and Characterization of Two Se6OMTs from Stephania epigaea Offer Novel Insights into the Biosynthetic Pathway of Cepharanthine
Source: Metabolites. 2025 Feb 3;15(2):92. doi: 10.3390/metabo15020092 (PMC11857138; doi:10.3390/metabo15020092)
Supplement: Supplementary file 1 [file metabolites-15-00092-s001.zip › metabolites-3411025-supplementary.pdf]

Supplementary data

Identification and Characterization of Two Se6OMTs from *Stephania epigaea* Offer Novel Insights into the Biosynthetic Pathway of Cepharanthine

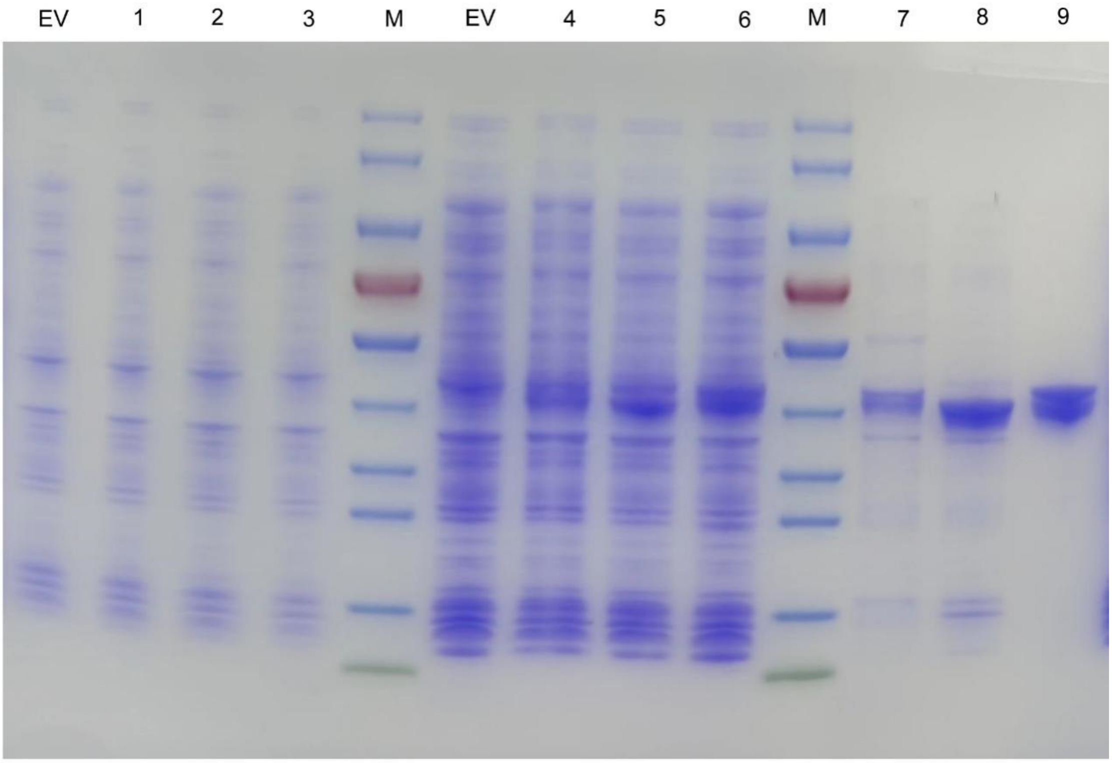

Figure S1. SDS-PAGE analyses was conducted on the purified proteins of St6OMT1-3 from the induced expression of the recombinant plasmid. Lane M: protein ladder, with molecular weights from top to bottom at 180, 130, 100, 70, 55, 40, 35, 25, 15, 10 kDa. Lanes 1-3: cell lysate before induction of St6OMT1-3, respectively. Lanes 2: the supernatant of induced cell lysate of St6OMT1-3. Lanes 3: the purified enzymes of St6OMT1-3. EV denotes the empty vector.

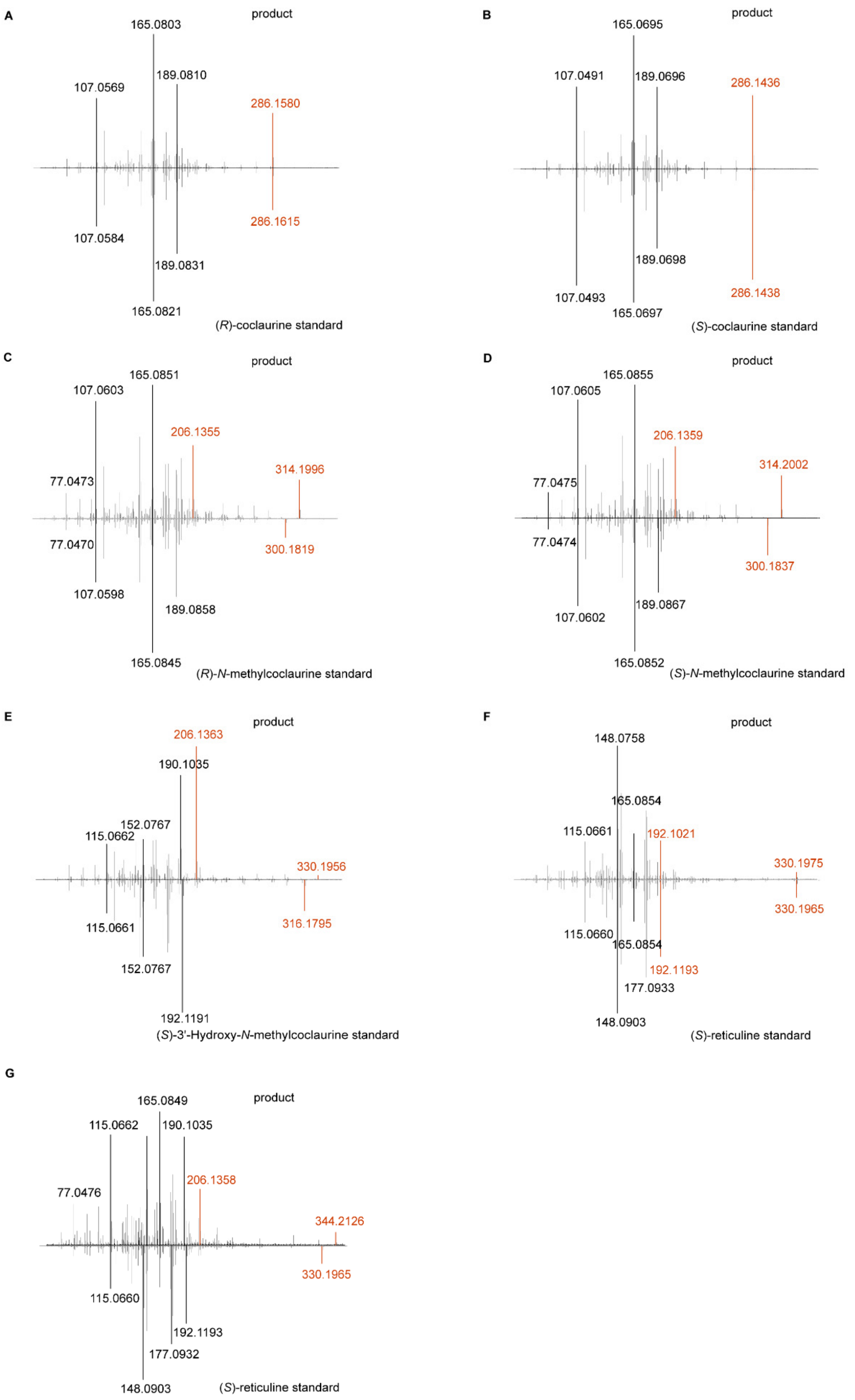

Figure S2. High-Resolution MS/MS spectrum fragmentation of products catalyzed by Se6OMTs. **A, B,** Mass spectrum of fragment ions of standard (*R*)- and (*S*)-coclaurine (downside) and reaction products of Se6OMT1 and Se6OMT3 with substrate (*R*)- and (*S*)-coclaurine(upside), respectively. **C**, **D**, Mass spectrum of fragment ions for standard (*R*)- and (*S*)- NMC are depicted in lower side, while the reaction products of Se6OMT3 with substrates (*R*)- and (*S*)-NMC are shown in upper side, separately. **E**, the comparison between (*S*)-HNMC standard and the products of Se6OMT3 with (*S*)-HNMC as the substrate. **F**, **G**, the comparison between (*S*)- reticuline standard and two BIAs produced by Se6OMT3 catalyzing (*S*)-HNMC and (*S*)-reticuline, respectively.

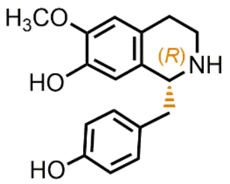

(*R*)-Coclaurine

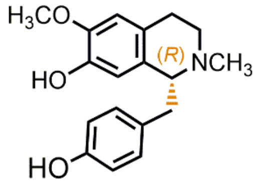

(*R*)-*N*-methylcoclaurine

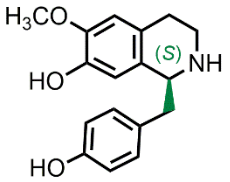

(*S*)-Coclaurine

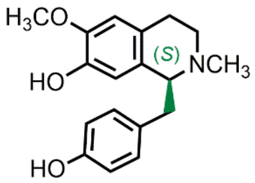

(*S*)-*N*-methylcoclaurine

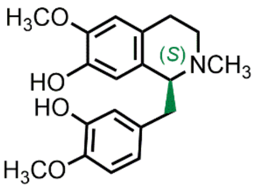

(*S*)-Reticuline

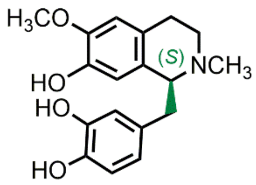

(*S*)-3'-Hydroxy-*N*-methylcoclaurine

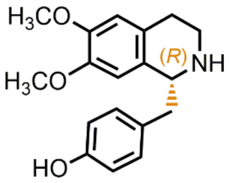

(*R*)-norarmepavine

Figure S3. Seven compound structures of 1-BIAs were tested as potential substrates of Se6OMTs.

Table S1. The full-length nucleic acid sequences of *Stephania epigaea* OMTs in this study.

gene	sequences
Se6OMT1	ATGGCATCAGAAAATCTAAAGCAAGACCAAGCAGCCCAAGCCAAGTTATGGAGCTTCATA-<br>TATGGCTTTGCAGAGTCACTAGTCGTCAAGTCTGTTATTCAGCTTGACATACCTGATATCATTCAC<br>AACCATGGCAAGCCCATCTCCCTTTCCAACCTTGCTTCTCGAATTCCTTCTCCGAAC-<br>GGCGCCGTGGATCACGACCGCTTGTTCCGGATCATGCGGTATTTGGTGCACATGAAGCTCTTCA<br>CGAAAGAGTCTATCGACGGCGAAGATCATTATGGTCTTGCGCCGGCCGCGAAGTACCTAG-<br>TGAAGGGGTGGGAGAAGAGCATGGTGCCTTCAATTCTATGTATAACTGATAAGGAGTTCATGTC<br>ACCATGGTACCATCTCAAGGAGGGGTTGGGGAACGAGAGCACGACAGCGTTCGA-<br>GAAGGCGTTGGGGATGAACATATGGGATTACATGTCGAAGAACCCGAAGAAGAACAACATCTT<br>CAATGAGGCCATGGCTTGTGACACTAGGCTACTCATGTCTGCATTGGTGAG-<br>TGAATGCAAGGACATGTTTCAAGGGATTAAGTCGCTAGTCGATGTCGGCGGGGGACTGGAACT<br>GCAGTTAGGCACATTGCCAAGGCCTACCCTCACATAAATTGCAGTGTC-<br>TATGATCTCCCTCATGTTGCTGCTGATTCTCCTACTTACCCTGAAGTGACTCGCATCGAAGGCGA<br>CATGTTCAAGTTCATTCCCAAAGCAGATGCCATCCTAATGAAGTG-<br>CATCCTTCATGACTGGGCCGACGACGAATGCATTAAGATTCTAAAGAAGTGCAAGGAAGCGTTG<br>CCGAGTAATGGGGGGAAAGTAATCCTCGTCGAGGTCGTGTTGAATGCGCTTTCGGAG-<br>CATCCATACACAAAGATAAGGATGGTGAGTGACTTGGACATGATGCTGAACTGTGGAGGTAAAG<br>AAAGAACAGAGGGGGAGTGGAAGAACCTCATCTATGCAGCTGGTTTTAGCAGGTACAA-<br>TATCAGGCAACTGGATTTCACACAGTATGTTATTGAGGTCTACCCTTGA
Se6OMT2	ATGGCAGCAGAAAATCTAGAGCAACACCAAGCAGCCCAAGCCAAGCTATGGAGC-<br>TACATCTATGGCTATACAGAGTCACTAGTACTCAAGTCTGCTGTTCAGCTTGACATCCCTGATATC<br>ATCCACAACCATGGCAAGCCCATCTCCCTCTCCGACCTTGCTCGCCGGATTTCCTCACCAA-<br>GCGGCGCCGTGGATCACAACCGCTTGTATCGGATCATGCGCTACTTGGTGTACATGAAGTTCTTC<br>ACGAAGGAGTCCGACGTCAATGGTGAAGATCGATATGGGCTTGCACCGCCCGCAAAGTAC-<br>CTAGTGAAGGGGTGGGAGAAGAGCATGGTACCTTCAATTCTAAGCCTCACTGATGAGGAATTCA<br>TGTTACCATGGTACCATCTCAAGGATGGTTTGTCGAACAATGGCACAACCGCGTTAGA-<br>GAAGGCGCTAGGGATGGGCATATGGGAATACATGTCGAAGAACCCCAAGACGAACAGCATCTT<br>CAATGAGGCCATGGCTTGTGACACTAGGCTGGTCATGTCTGCATTGGTGAG-<br>TGAATGCAAGGACATGTTTCAAGGAATTAAGTCACTAGTCGATGTCGGCGGGGGCACTGGGACT<br>GCTGTTAGGTACATTGCCAAGGCCTTCCCTTACATAAATTGCACTGTC-<br>TATGATCTCCCTCATGTTGCTGCTGATTCTCTTACTTACCCCGAAGTGACTCGCGTCGAAGGCGA<br>CATGTTCAAATACATTCCTAAAGCTGATGCCATCCTAATGAAGTACATTC-<br>TACATGACTGGGCCGACGAGGAAAGCATTCAGATACTGAAGAAGTGTAAGGAAGCATTGCCGA<br>GTGATGGGGGGAAGGTAATCCTAGTCGATATCGTGTTGAATCCGTTTTCGGAG-<br>CATCCATACGCGAAGGTGAGGATGGTGAGCGACGTGGACATGATGCAGAGCTGTGGAGGGAAA<br>GAGAGGACAGAGGAGGAGTGGAAGAAGCTCATCTATGCTGCAGGTTTTAG-<br>TAGGTACAACATTAGGCACATAAATACCATACCATCTGTTATTGAGGTTTTTCCTTGA
Se6OMT3	ATGGCAACACAAAATCTAAAGCAAGACCAAGCATCCCAAGCAAAGCTATGGAGCTTCATA-<br>TATGGCTTTGCAGAGTCACTAGTCCTCAAGTCTGCCATTCAACTTGACATACCTGATATCATTCAC<br>AACCATGGCAAGCCCATGACCCTATCCAACCTTGCTTCTCGAATTCCCTCTCCGAAC-<br>GGCGCCGCGGATCACGACCGCTTGTTCCGGATCATGCGCTATTTGGTGCACATGAATCTCTTCAC<br>GAAAGAGTCTATCGACGGCGAAGATCATTATGGTCTTGCGCCGGCCGCGAAGTACCTAG-<br>TGAAGGGGTGGGAGAAGAGCATGGTGCCTTCAATTCTATGTATAACTGATAAGGAGTTCATGTC<br>ACCATGGTACCATCTCAAGGAGGGGTTGGGGAACGAGAGCACGACAGCGTTCGA-<br>GAAGGCGTTGGGGATGAACATATGGGATTACATGTCGAAGAACCCGAAGAAGAACAACATCTT<br>CAATGAGGCCATGGCTTGTGACACTAGGCTACTCATGTCTGCATTGGTGAG-<br>TGAATGCAAGGACATGTTTCAAGGGATTAAGTCGCTAGTCGATGTCGGCGGGGGACTGGAACT<br>GCAGTTAGGCACATTGCCAAGGCCTACCCTCACATAAATTGCAGTGTC-<br>TATGATCTCCCTCATGTTGCTGCTGATTCTCCTACTTACCCCGAAGTGACTCGCATCGAAGGCGA<br>CATGTTCAAGTTCATTCCCAAAGCAGATGCCATCCTAATGAAGTG-<br>CATCCTTCATGACTGGGCCGACGACGAATGCATTAAGATTCTAAAGAAGTGCAAGGAAGCGTTG<br>CCGAGTAACGGGGGGAAGGTAATCCTAGTCGATATCGTGTTGAATGCGTTTTCGGAG-<br>CATCCATACACGAAGATGAGGATGGTGATGGACTTGGACATGATGCTGAACTGTGGAGGGAAA<br>GAGAGGACAGAGGAGGAGTGGAAGAAGCTCATCTATGCTGCAGGTTTCAGTAGGTACAA-<br>GATTAGGCAAGTGAATGCCATACAGTCTGTTATTGAGGTTTACCCTTACTGA

Table S2. The primer sequences for cloning Se6OMTs-pET-28a.

gene	PET-28a primer sequences
Se6OMT1-F	CAGCAAATGGGTCGCGGATCCATGGCATCAGAAAATCTAAAGCAA
Se6OMT1-R	ACGGAGCTCGAATTCGGATCCTCAAGGGTAGACCTCAATAACAT
Se6OMT2-F	CAGCAAATGGGTCGCGGATCCATGGCAGCAGAAAATCTAGAG
Se6OMT2-R	ACGGAGCTCGAATTCGGATCCTCAAGGAAAAACCTCAATAACAGATG
Se6OMT3-F	CAGCAAATGGGTCGCGGATCCATGGCAACACAAAATCTAAAGCA
Se6OMT3-R	ACGGAGCTCGAATTCGGATCCTCAGTAAGGGTAAACCTCAATAACA

Table S3. The Physical and chemical properties of Se6OMTs in this study.

**protein**	**Se6OMT1**	**Se6OMT2**	**Se6OMT3**
Molecular weight	39406.59	39605.66	39645.02
Theoretical pI	6.27	6.02	6.60
Total number of negatively charged residues	43	44	43
Total number of positively charged residues	39	39	41
Formula	$C_{1768}H_{2767}N_{463}O_{510}S_{23}$	$C_{1783}H_{2780}N_{460}O_{519}S_{20}$	$C_{1776}H_{2785}N_{465}O_{510}S_{26}$
Total number of atoms	5531	5562	5562
Instability index	41.64	40.76	42.82
Aliphatic index	89.74	88.63	86.47
Grand average of hydropathicity	-0.156	-0.183	-0.184

Table S4. Abbreviations and GenBank accessions numbers for functionally characterized plants OMTs used for phylogenetic analysis.

Gene	Source	Accession
Ps6OMT	*Papaver somniferum*	AAP45315
Ps4'OMT	*Papaver somniferum*	AAP45313
Ps7OMT	*Papaver somniferum*	AAQ01668
PsNMT4	*Papaver somniferum*	AOR51553
PsTNMT	*Papaver somniferum*	Q108P1
Cj6OMT	*Coptis japonica*	BAB08004
St6OMT	*Stephania tetrandra*	Reference[20]
Cj4'OMT	*Coptis japonica*	BAB08005
CjSOMT	*Coptis japonica*	BAA06192
CjCoOMT	*Coptis japonica*	BAC22084
CjCNMT	*Coptis japonica*	BAB71802
GflOMT7	*Glaucium flavum*	AKO60158
NnOMT5	*Nelumbo nucifera*	XP_010276063
SiCNMT1	*Stephania intermedia*	MK749412
SiCNMT2	*Stephania intermedia*	MK749413
SiCNMT3	*Stephania intermedia*	MK749414
MsChOMT	*Medicago sativa*	AAB48059
Cc6OMT1	*Coptis chinensis*	AXC09385
